# Physiological and Phytochemical Responses to Cadmium Stress in Three Wheat Cultivars

**DOI:** 10.3390/biology15211932

**Published:** 2026-10-24

**Authors:** Laura Židek Jozefíková, Anirban Jyoti Debnath, Martina Gregorová, Peter Nemeček, Petra Ranušová, Monika Šutáková, Miroslava Sinčák, Pavol Hauptvogel, Ildikó Matušíková

**Affiliations:** 1 Institute of Chemistry and Environmental Sciences, University of Ss. Cyril and Methodius in Trnava, Nám. J. Herdu 2, 917 01 Trnava, Slovakia; laura.zidekova@gmail.com (L.Ž.J.); anirbandebnath@ymail.com (A.J.D.); martina.gregorova4@gmail.com (M.G.); peter.nemecek@ucm.sk (P.N.); pranusova@gmail.com (P.R.); monika.sutakova@ucm.sk (M.Š.); ildiko.matusikova@ucm.sk (I.M.); 2 Faculty of Forestry and Wood Sciences, Czech University of Life Sciences Prague, Kamycka 129, Praha 6—Suchdol, 16500 Prague, Czech Republic; 3 Research Institute of Plant Production, National Agricultural and Food Centre, Bratislavská cesta 122, 921 68 Piešťany, Slovakia; p.hauptvogel@radosina.sk

**Keywords:** cadmium, wheat cultivars, cultivar-specific responses, phenolic metabolism, rutin, pathogenesis-related proteins, subcellular partitioning, phenolic profiling

## Abstract

Wheat cultivars differ markedly in their responses to cadmium (Cd), but it remains unclear how these differences are reflected across physiological and biochemical levels. This study investigated the physiological, enzymatic, and phenolic responses of three Slovak wheat cultivars representing different genomic backgrounds (tetraploid emmer wheat Zirnitra and hexaploid bread wheat Sunanka, and Ilona) exposed to 50 mg L^−1^ Cd^2+^ (as CdCl_2_) for 10 days, integrating growth and pigment responses with Cd accumulation, subcellular partitioning, pathogenesis-related (PR) enzyme activities, and targeted profiling of phenolic metabolites. Cd similarly reduced shoot fresh weight in all cultivars, while chlorophyll content declined significantly in Sunanka and Ilona but remained stable in Zirnitra. All cultivars strongly restricted root-to-shoot Cd translocation, with Cd predominantly recovered in the combined cytosolic/vacuolar fraction. The integrated analysis revealed particularly pronounced cultivar-specific differences in phenolic profiles. Sunanka shoots showed a reciprocal rutin (RUT)–quercetin (QUER) response, with RUT becoming undetectable and QUER strongly accumulating, whereas Ilona roots showed depletion of trans-ferulic acid and chlorogenic acid accompanied by marked protocatechuic acid accumulation. In Zirnitra roots, RUT increased markedly, and root RUT was positively associated with Cd accumulation after FDR correction. PR-enzyme activities also responded in a cultivar-, tissue-, and activity-band-specific manner. Overall, the findings demonstrate that similar growth inhibition and restricted Cd translocation can coexist with distinct biochemical responses in wheat cultivars of different genomic backgrounds, highlighting root rutin accumulation as a candidate for further investigation in relation to Cd exposure.

## Simple Summary

Cadmium is a toxic metal that can accumulate in wheat and adversely affect plant growth. We compared three wheat cultivars to examine their physiological and biochemical responses to cadmium. Although all cultivars retained most cadmium in the roots and showed similar reductions in shoot growth, they differed markedly in photosynthetic pigments, phenolic compounds, and defence-related enzyme activities. Rutin, a naturally occurring plant flavonoid, showed a particularly distinct response, with root rutin concentration positively associated with cadmium accumulation across cultivars. Overall, the study reveals differences in biochemical responses to cadmium among wheat cultivars despite broadly similar effects on growth. Species- and ploidy-related effects cannot be distinguished from cultivar-specific responses.

## 1. Introduction

Cadmium (Cd) is a non-essential and highly toxic trace element that can accumulate in agricultural soils and be readily taken up by crop plants. In wheat (*Triticum* spp.), Cd exposure can impair plant growth and photosynthetic performance, including chlorophyll biosynthesis and stability [1,2], while its accumulation in edible tissues represents an additional concern because of its potential entry into the human food chain [3]. Importantly, wheat genotypes can differ substantially in Cd accumulation and distribution within the plant [4,5,6]. Understanding the mechanisms underlying this genotypic variation is therefore relevant both to plant responses to Cd stress and to efforts aimed at limiting Cd accumulation in aboveground tissues.

Wheat (*Triticum* spp.) is one of the world’s major cereal crops and an important contributor to global food security [3]. Bread wheat (*Triticum aestivum* L.) is hexaploid (AABBDD, 2n = 6x = 42), whereas emmer wheat (*Triticum turgidum* ssp. *dicoccum*) is tetraploid (AABB, 2n = 4x = 28), reflecting differences in their genomic constitution. Cadmium contamination is of particular concern in wheat because this non-essential toxic metal can be taken up from contaminated soils and accumulate in wheat tissues [1,4,5], potentially affecting crop productivity and food safety. Understanding Cd uptake, distribution, and associated physiological and metabolic responses in wheat is therefore relevant to both crop production and food safety.

Wheat plants employ multiple, partly overlapping mechanisms to limit Cd toxicity, including restriction of Cd uptake and root-to-shoot transport, retention in root tissues, binding to cell-wall components, and intracellular sequestration [4,5,7]. Subcellular compartmentation can contribute to Cd detoxification by restricting its interaction with sensitive cellular processes. In wheat, Cd has been shown to accumulate predominantly in the combined cytosolic/vacuolar fraction [8], although predominant accumulation in the cell-wall fraction, particularly in roots, has also been reported [7], indicating that subcellular Cd partitioning may vary among genotypes and experimental conditions. Importantly, the contribution and coordination of these protective processes are genotype dependent: comparative physiological, metabolomic, transcriptomic, and proteomic studies have revealed substantial differences in Cd accumulation and stress responses among wheat genotypes [4,5,6]. Cd tolerance and accumulation in wheat are better viewed as outcomes of multiple interacting processes rather than a single uniform mechanism.

Beyond mechanisms controlling Cd uptake, translocation, and compartmentation, Cd tolerance involves extensive biochemical adjustments aimed at limiting metal reactivity and maintaining cellular homeostasis. These include chelation by thiol-containing compounds such as glutathione and phytochelatins, regulation of cellular redox balance through enzymatic antioxidant systems, and changes in non-enzymatic antioxidants and other protective metabolites [8,9,10]. Cd exposure can also alter primary and secondary metabolism, stress-related signalling, and the expression or activity of defence-associated proteins [6,11,12,13]. Importantly, these responses are interconnected rather than acting independently: metal chelation, redox regulation, metabolic adjustment, and cellular sequestration collectively determine the biochemical consequences of Cd exposure [14,15]. Their relative contribution may differ among genotypes, providing multiple biochemical routes through which plants can respond to a similar Cd challenge.

Within this broader biochemical response, phenolic metabolism is of particular interest because phenolic compounds constitute a structurally and functionally diverse group of metabolites involved in plant–environment interactions and stress responses [16]. The shikimate pathway and the downstream phenylpropanoid network give rise to a broad spectrum of phenolic metabolites, including hydroxycinnamic and hydroxybenzoic acids and flavonoids, although individual compounds may originate through distinct and interconnected biosynthetic routes [16,17]. Evidence that Cd affects phenolic metabolism in wheat is already available. Targeted analyses have demonstrated Cd-dependent changes in individual phenolic acids and flavonoids, with responses varying with Cd concentration and experimental conditions [18], whereas integrated metabolomic and transcriptomic analyses identified phenylpropanoid metabolism as a major Cd-responsive pathway and revealed significant increases in caffeic and ferulic acid levels [19]. However, current evidence remains fragmented across individual metabolites, experimental conditions, and analytical approaches, providing a limited picture of how different components of the phenolic network are co-ordinately reorganized within the same biological system. In particular, comparative information on tissue- and genotype-dependent responses of individual phenolic metabolites, and their relationship to Cd accumulation, remains limited in wheat.

A complementary component of the biochemical response to Cd involves pathogenesis-related (PR) proteins, although these proteins have been studied primarily in the context of plant defence against pathogens [20]. Chitinases (PR-3) and β-1,3-glucanases (PR-2), in particular, have been reported to respond to Cd and other metals in cereals and other plant species [21,22,23]. Whereas low-molecular-weight metabolites can contribute directly to metal chelation and redox homeostasis, PR proteins may represent a functionally distinct component of the stress response. Chitinases can contribute to the generation of signalling-active oligosaccharides, while β-1,3-glucanases participate in callose turnover at plasmodesmata and may thereby influence cell-to-cell communication and stress signalling [21,24]. Although their responsiveness to metal exposure is well documented, the functional significance of these responses under metal stress remains less clearly resolved. PR-enzyme responses can vary among genotypes, tissues, enzyme forms, and metal treatments [21,23,24,25], while their relationships with tissue metal accumulation and other components of the biochemical stress response remain poorly understood. Integrating PR-enzyme responses with Cd accumulation, subcellular partitioning, and metabolic changes may therefore provide a more comprehensive view of whether different wheat genotypes respond to Cd through common or distinct biochemical adjustments.

Although metabolomic and transcriptomic studies have revealed broad Cd-responsive metabolic changes in wheat [4,19], comparatively little is known about the coordinated responses of individual phenolic metabolites across interconnected branches of shikimate/phenylpropanoid metabolism, particularly across genotypes and tissues. We therefore compared the hexaploid bread wheat cultivars Ilona and Sunanka with the tetraploid emmer wheat cultivar Zirnitra. We hypothesized that Cd exposure would induce both shared and cultivar-dependent physiological and biochemical responses, with changes in phenolic profiles representing a prominent component of the stress response. Accordingly, we combined Cd accumulation and partitioning with physiological responses, targeted phenolic profiling, and PR-enzyme activities.

## 2. Materials and Methods

### 2.1. Plant Material and Cadmium Treatment

Seeds of wheat (*Triticum aestivum* L.; cultivars Sunanka and Ilona) and emmer wheat (*Triticum dicoccum* L.; cultivar Zirnitra) were obtained from the Gene Bank of the Slovak Republic (Research Institute of Plant Production, Piešťany, NPPC Lužianky). Passport data are available via the Genetic Resources Information System of Slovakia (GRISS, https://griss.vurv.sk (accessed on 20 October 2026); accession no. SVK001 C01 05229 for Sunanka, SVKPIEST 1637 for Ilona, and SVK001 C01 05341 for Zirnitra).

Seeds were surface-sterilised in 5% (*v*/*v*) sodium hypochlorite (Centralchem, Banská Bystrica, Slovakia) for 10 min and rinsed three times with sterile distilled water. They were germinated on two layers of sterile filter paper moistened with distilled water in sterile plastic Petri dishes (90 × 15 mm) in a growth chamber (model KBWF 720, BINDER GmbH, Tuttlingen, Germany) at 24/18 ± 2 °C in the dark. After 3 days, seedlings were transferred to sterile plastic containers (10 × 10 × 16 cm) containing 500 mL of 25% Hoagland nutrient solution prepared according to [26] and supported on floating polystyrene platforms, with roots extending into the nutrient solution and shoots remaining above its surface. They were cultivated under controlled conditions (24/18 ± 2 °C, 60% relative humidity, 16/8 h photoperiod, light intensity approximately 210 µmol m^−2^ s^−1^). The nutrient solution was not renewed during the 10-day experimental period, 50 mg L^−1^ represents the nominal initial Cd concentration rather than a verified constant exposure level.

The experiment was arranged in a completely randomised block design with three biological replicates per treatment, each represented by an individual cultivation container. Each container contained 3 plants to provide sufficient material for the individual analyses; plants within the same container were not considered independent biological replicates. No a priori power analysis was performed; therefore, the sample size may have limited the detection of smaller treatment or cultivar effects. For individual analyses, plant material was sampled randomly from each biological replicate according to the requirements of the respective assay. Tissues were either analysed individually or tissues from three individual plants were pooled when necessary. Where plant tissues were pooled, one pooled sample per cultivation container represented one biological replicate. After 10 days of cultivation, plants were transferred to fresh 25% Hoagland solution supplemented with 50 mg L^−1^ Cd supplied as CdCl_2_ (Merck, Darmstadt, Germany). The Cd concentration (50 mg L^−1^) was selected in accordance with previous studies on cadmium responses in wheat [27,28], soybean [27,28] and oat [22], in which this dose reliably induced a measurable stress response without causing complete growth arrest or plant death within a comparable exposure window. Using a consistent Cd dose across this broader research programme also allows direct comparison of cultivar- and species-specific responses. Control plants were maintained in Hoagland solution without Cd addition. The cadmium treatment lasted 10 days, and the nutrient solution was not renewed during this period. At the end of the treatment, fresh plant material was harvested for further analyses. Samples intended for analyses requiring preserved or dry material were immediately processed or stored under the conditions specified below for the respective assay.

### 2.2. Growth Parameters and Photosynthetic Pigments

Shoot and root fresh weight (FW) was determined in one randomly selected plant from each biological replicate (container) immediately after harvest. Plant tissues were gently blotted dry with filter paper and weighed using an analytical balance (model ABS 320-4N, KERN & SOHN GmbH, Balingen, Germany).

Photosynthetic pigments were determined according to Lichtenthaler and Wellburn [29]. Shoot tissue (50 mg), collected from one randomly selected plant from each biological replicate, was homogenised with sea sand in 5 mL of 80% (*v*/*v*) acetone (Centralchem) and centrifuged at 6000× *g* for 10 min at 4 °C. The absorbance of the supernatant was measured at 663, 646, and 470 nm using a spectrophotometer (Cary 50 UV–Vis, Varian Inc., Palo Alto, CA, USA). Pigment contents (chlorophyll *a*, chlorophyll *b*, total chlorophyll, and carotenoids) were calculated according to the cited method and expressed as mg g^−1^ fresh weight.

### 2.3. Determination of Cadmium Content and Subcellular Distribution

Cadmium (Cd) concentrations in shoots and roots were determined by atomic absorption spectrometry (AAS). Shoot and root tissues collected from 3 plants were pooled separately prior to analysis. One pooled shoot sample and one pooled root sample from each cultivation container therefore represented the corresponding biological replicate (*n* = 3 per cultivar and treatment). Samples were treated with 5 mM EDTA (Merck, Darmstadt, Germany) for 15 min to remove surface-bound Cd, rinsed twice with distilled water, and dried at 70 °C for 72 h in a hot air oven. Dried plant material (200 mg) was digested in 8 mL of concentrated HNO_3_ (Sigma-Aldrich, Darmstadt, Germany) using a microwave digestion system (Multiwave 3000, Anton Paar GmbH, Graz, Austria). Cadmium concentration was determined using an atomic absorption spectrometer (iCE 3000, Thermo Scientific, Waltham, MA, USA) with certified reference material ERM-CD281 (Merck, Germany) for quality control. The translocation factor (TF) was calculated as the ratio of Cd concentration in shoots to that in roots.

Subcellular distribution of Cd was analysed using the radioisotope ^109^Cd. Three-day-old seedlings were transferred to fresh Hoagland solution for 10 days. The medium was supplemented with ^109^Cd (Czech Metrological Institute, Brno, Czech Republic), corresponding to an activity of 800.4 Bq per 3 mL of medium (266.8 Bq mL^−1^), and CdCl_2_ was used as a carrier to obtain a final Cd concentration of 0.05 g L^−1^. The plants were exposed for 10 days. At harvest, shoot and root tissues from 3 selected plants were pooled separately for each biological replicate. Each pooled sample originated exclusively from plants grown within the same cultivation container; samples from different containers were not combined. Root samples were treated with 5 mM Na_2_-EDTA for 15 min to remove surface-bound Cd, rinsed with distilled water, frozen in liquid nitrogen, and homogenised in cold extraction buffer (50 mM Tris–HCl, 250 mM sucrose, 1 mM dithioerythritol; all from Merck). Subcellular fractionation was performed using differential centrifugation as previously described by Žideková et al. [9], yielding cell wall (CW), organellar (ORG), and cytosolic/vacuolar (CYT) fractions. Radioactivity of individual fractions was measured using a gamma spectrometer (76BP76/3, Scionix Holland B.V., Bunnik, The Netherlands) equipped with a well-type NaI(Tl) detector. Instrument calibration was performed using standard radionuclide solutions, and measurements were conducted under identical geometry conditions. The accumulation of ^109^Cd in individual fractions was expressed as Bq g^−1^ dry weight. Due to the logistical and regulatory constraints associated with radioisotope handling, the ^109^Cd subcellular distribution experiment was conducted as a single temporal run, whereas all other analyses were performed on independently cultivated biological replicates. Within this run, however, 3 independently cultivated containers per cultivar/treatment served as biological replicates. The relative distribution of ^109^Cd among subcellular fractions (cell wall, CW; organellar, ORG; cytosolic/vacuolar, CYT) was expressed as a percentage of the sum of radioactivity recovered in the three fractions, with the three fractions summing to 100% for each sample. As total radioactivity recovery was not independently assessed, potential losses during fractionation cannot be excluded.

### 2.4. Chitinase and Beta-1,3-Glucanase Activity

Total protein was extracted from 200 mg of shoot and root tissues. For each biological replicate, individual tissue from one randomly selected plant within the corresponding cultivation container was used. Samples were frozen in liquid nitrogen, homogenised (TissueLyser II, Qiagen, Germantown, MD, USA), and extracted in 0.1 M sodium acetate buffer (pH 5.2) containing 100 µM phenylmethylsulfonyl fluoride (all components from Merck). The homogenate was centrifuged at 13,000× *g* for 15 min at 4 °C, and the supernatant was collected and stored at −80 °C. Protein concentration was determined [30], and equal amounts of total protein (15 µg per lane) were loaded for electrophoresis. Proteins were separated by SDS-PAGE [31] using 12.5% polyacrylamide gels (Merck). Chitinase and β-1,3-glucanase activities were detected by substrate-specific zymography as described previously [21,23]. Briefly, gels were polymerised with glycol chitin or laminarin (Sigma-Aldrich ) for chitinase and β-1,3-glucanase detection, respectively. After electrophoresis, proteins were renatured in acetate buffer containing Triton X-100 (Sigma-Aldrich) and incubated under appropriate conditions to allow enzyme activity. Chitinase activity was visualised using Fluorescent Brightener 28 (Sigma-Aldrich) under UV light, while β-1,3-glucanase activity was detected using triphenyltetrazolium chloride staining. Gel images were recorded and analysed using ImageJ software version 1.54 (National Institutes of Health, Bethesda, MD, USA) [32]. For each biological replicate, all corresponding experimental variants were analysed on the same gel. Background subtraction was performed using the rolling-ball algorithm (radius 30 pixels), and band intensities were quantified as mean gray values within identically sized regions of interest (ROIs). No additional intensity normalization was performed after background correction. Activity bands were identified by their electrophoretic migration relative to a co-separated molecular weight standard, and corresponding bands were matched within each cultivar and tissue. Background-corrected mean densities were quantified separately for individual activity bands (MD, in pixels).

### 2.5. Determination of Phenolic Compounds by HPLC

The concentrations of rutin, quercetin, epicatechin, caffeic acid, chlorogenic acid, trans-ferulic acid, vanillic acid, gallic acid, and protocatechuic acid in the shoots and roots were determined using an HPLC method combined with an SPE-based extract purification step, as described by Ranušová et al. [33].

For each biological replicate, shoot or root material from three plants sampled from the corresponding cultivation container was pooled, immediately frozen in liquid nitrogen, and freeze-dried (−55 °C; approximately 10 Pa; 24 h) (Lyovapor L-200, BÜCHI Labortechnik AG, Flawil, Switzerland). One pooled sample per container represented one biological replicate (*n* = 3 per cultivar and treatment). Freeze-dried plant material was homogenised, and phenolic compounds were extracted from 100 mg dry weight using 80% (*v*/*v*) methanol with ultrasound-assisted extraction for 20 min. Extracts were centrifuged (13,000× *g*, 10 min), filtered (0.45 μm), and evaporated under reduced pressure. The residues were reconstituted and subjected to SPE purification [33]. Chromatographic separation was performed using an HPLC system (YL9100, Young Lin Instrument Co., Ltd., Anyang, South Korea) equipped with a diode array detector. Separation was achieved on a C18 column (150 × 4.6 mm, 3 μm) at 35 °C with a flow rate of 1 mL min^−1^ and an injection volume of 5 μL. The mobile phase consisted of acidified water and acetonitrile, and a gradient elution program was applied [33]. Detection was carried out at multiple wavelengths (240–350 nm), and compounds were identified based on retention times and UV spectra compared to standards. Quantification was performed using external calibration curves.

### 2.6. Statistical Analysis

Data were analysed by one-way analysis of variance (ANOVA), with the six genotype–treatment combinations treated as levels of a single factor. Differences among means were evaluated using Duncan’s multiple range test at *p* < 0.05 (IBM SPSS Statistics 19). Individual PR-enzyme bands were compared between control and Cd-treated samples within each cultivar and tissue using two-sided Welch’s t-tests, followed by Benjamini–Hochberg FDR correction (q < 0.05). Cross-cultivar comparisons of individual bands were avoided due to differences in enzyme activity profiles. In addition to one-way ANOVA, two-way ANOVA was performed separately for roots and shoots to assess cultivar, Cd treatment, and their interaction, allowing direct evaluation of genotype-dependent Cd responses. Given the limited biological replication (*n* = 3 per group), multiple testing, and cultivar-specific PR-enzyme band profiles, the results were interpreted cautiously and used primarily as complementary evidence. Complete ANOVA and FDR-adjusted enzyme activity results are available in the Supplementary Data S1 deposited in Zenodo. ^109^Cd subcellular distribution was analysed separately in shoots and roots using a mixed-effects model with genotype, subcellular fraction (CW, ORG, and CYT), and their interaction as fixed effects and biological replicate as a random effect. Post hoc comparisons among genotypes within fractions were performed at *p* < 0.05 (*n* = 3).

PR-enzyme activities were analysed separately using Student’s t-test, comparing control and Cd-treated samples within each cultivar, tissue, and activity band. Only activity bands quantifiable under both treatments were statistically evaluated.

Relationships among measured parameters were assessed using Spearman’s rank correlation analysis in JMP 11 (SAS Institute Inc., Cary, NC, USA). The analysis included 18 biological samples per tissue (three cultivars × two treatments × three replicates). Subsequently, the *p*-values were adjusted for multiple testing using the Benjamini–Hochberg false discovery rate (FDR), with q < 0.05 considered significant. FDR correction was applied separately within each tissue after excluding correlation pairs involving variables not detected in all three cultivars of the corresponding tissue. The FDR correction included 45 tests for roots and 105 tests for shoots. Undetected values were recorded as zero; no observations were missing from the correlation dataset. Principal component (PC) analysis was performed separately for shoot and root datasets using physiological parameters and metabolite concentrations. The analyses included 18 biological samples per tissue, and the complete set of variables is provided in Supplementary Data S2. PR-enzyme activities were excluded from the PCA because their activity-band profiles differed markedly among cultivars, resulting in a variable set of bands that could not be consistently compared across all cultivars. For correlation analysis, PCA and HCA, metabolites with no detectable signal were assigned a value of zero. All variables were standardized prior to PC analysis, and for graphical clarity, only loading vectors corresponding to variables showing significant effects in univariate analyses were displayed.

Hierarchical cluster analysis (HCA) was performed using the same variables as PCA with Ward’s method and squared Euclidean distance, as a complementary assessment of sample grouping. Both PCA and HCA were used as exploratory analyses to visualize similarities and differences among samples, rather than as independent validation of the observed treatment effects. JMP 11 was used for PCA and HCA.

## 3. Results

### 3.1. Cadmium Inhibits Shoot Growth and Differentially Affects Photosynthetic Pigments Among Wheat Cultivars

**Figure 1:**
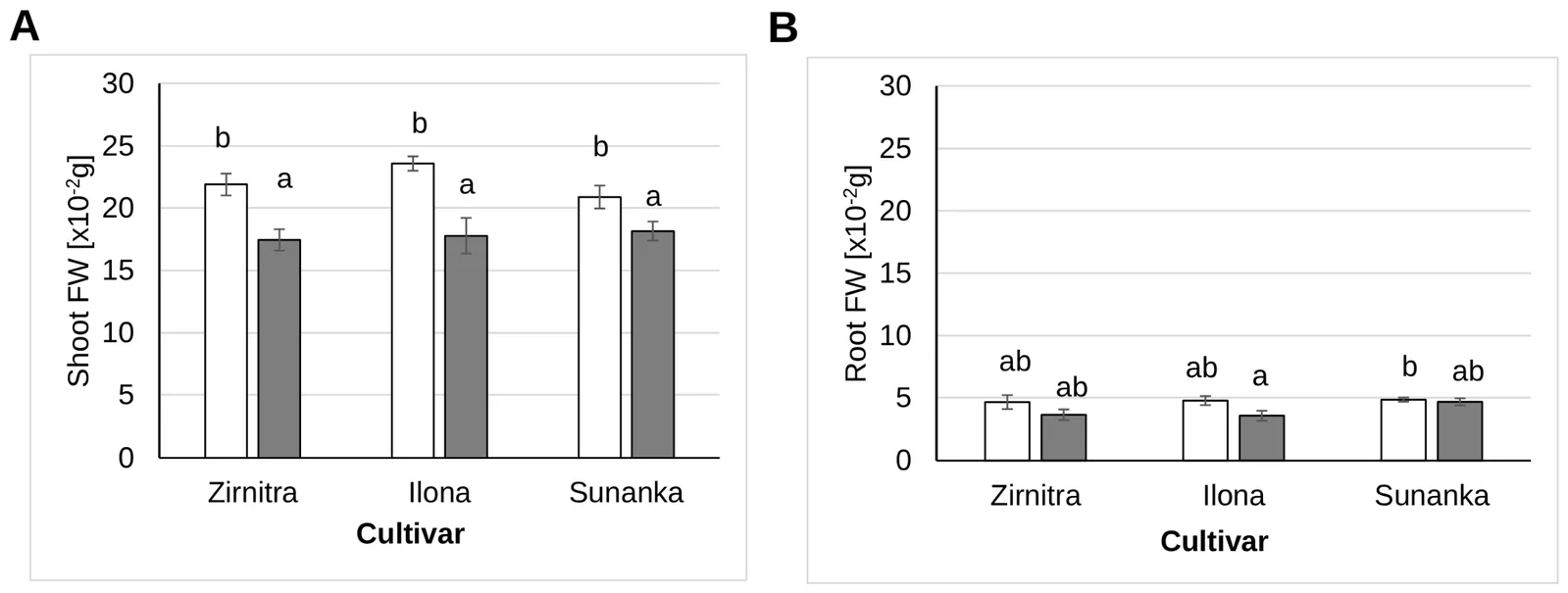
Effect of Cd treatment on shoot and root fresh weight of three wheat cultivars. Shoot (**A**) and root (**B**) fresh weight (FW) of Zirnitra, Sunanka, and Ilona under control and Cd treatment. White bars represent control plants, and grey bars represent Cd-treated plants. Data are means ± SD (*n* = 3 biological replicates). Different letters above the bars indicate significant differences among cultivar × treatment combinations (one-way ANOVA followed by Duncan’s multiple range test, *p* < 0.05).

Exposure to 50 mg L^−1^ Cd^2+^ for 10 days significantly reduced shoot fresh weight in all three cultivars (Figure 1A). Root fresh weight was significantly reduced only in Ilona, while Sunanka and Zirnitra showed no statistically significant change (Figure 1B). Two-way ANOVA revealed no significant cultivar × Cd interaction for fresh weight in either organ (Supplementary Data S1).

**Figure 2:**
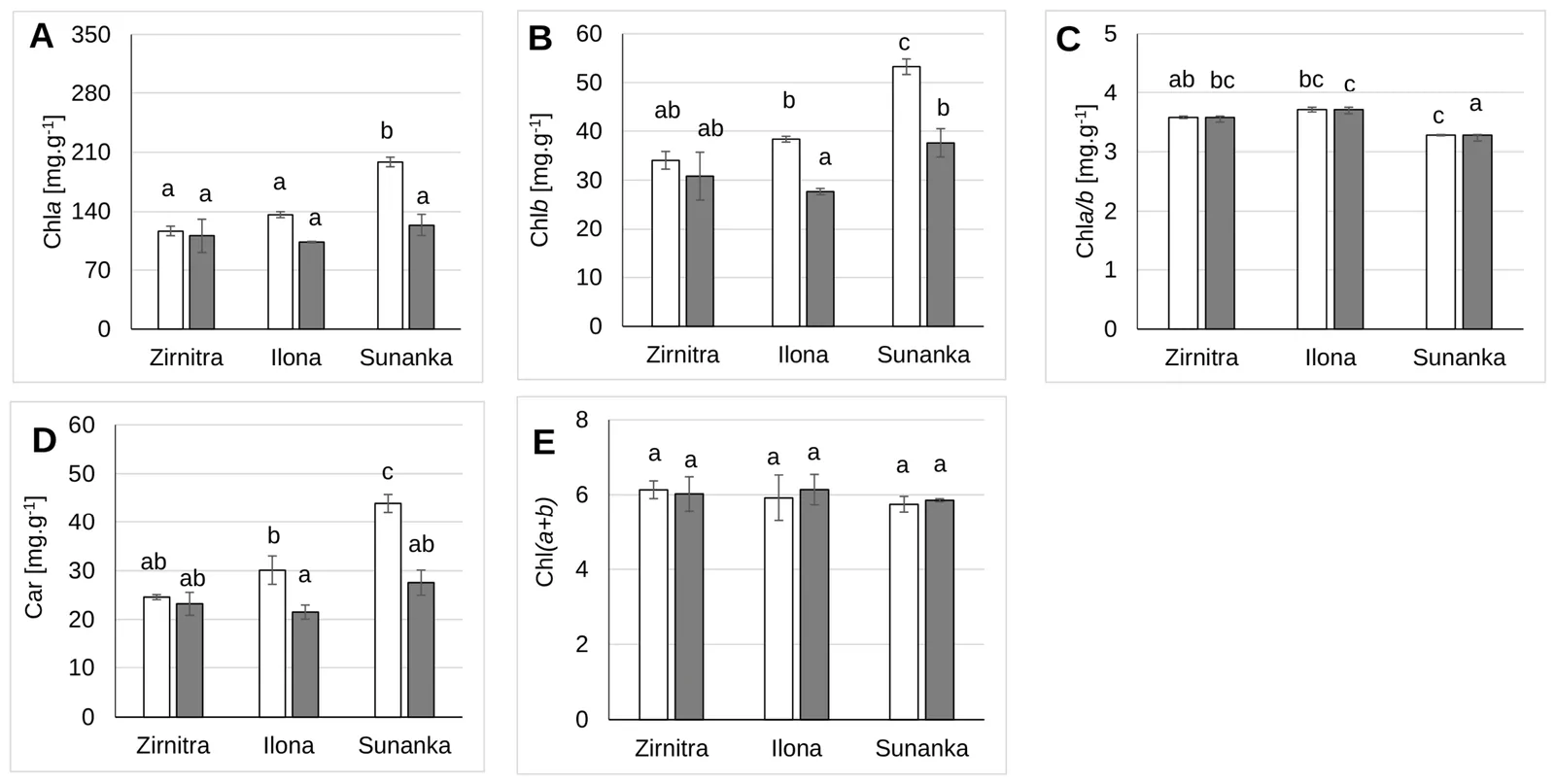
Photosynthetic pigment content in three wheat cultivars under control and Cd treatment. (**A**) Chlorophyll *a*, (**B**) chlorophyll *b*, (**C**) chlorophyll *a*/*b* ratio, (**D**) carotenoids, and (**E**) total chlorophyll-to-carotenoid ratio (Chl (*a*+*b)*/Car). White bars represent controls and grey bars Cd-treated plants. Data are means ± SD (*n* = 3 biological replicates). Different letters above the bars indicate significant differences among cultivar × treatment combinations (one-way ANOVA followed by Duncan’s multiple range test, *p* < 0.05).

Shoot pigment contents and their ratios showed cultivar-specific differences under control conditions and in response to Cd treatment (Figure 2). Sunanka exhibited significantly higher contents of chlorophyll *a*, chlorophyll *b*, and carotenoids compared to Zirnitra and Ilona, demonstrating cultivar-specific differences in constitutive pigment levels. Cadmium treatment significantly reduced pigment contents in Sunanka and Ilona, while Zirnitra showed no statistically significant changes (Figure 2A,B,D). Despite these reductions, pigment levels after Cd treatment remained comparable among the cultivars, except for chlorophyll B, which remained significantly lower in Ilona than in Sunanka. The Chl*a*/*b* ratio decreased significantly only in Sunanka (Figure 2C), indicating a proportionally greater reduction in chlorophyll *a* than chlorophyll *b*. In contrast, the total chlorophyll-to-carotenoid ratio (Chl(*a*+*b*)/Car) remained stable across all cultivars (Figure 2E), indicating that Cd did not markedly alter the relative proportion of total chlorophylls to carotenoids. Significant cultivar × Cd interactions were detected for chlorophyll *a*, carotenoids, the chlorophyll *a*/*b* ratio, and total chlorophyll content (Supplementary Data S1).

### 3.2. Cadmium Is Predominantly Retained in Roots and Accumulates Mainly in the Cytosolic/Vacuolar Fraction

**Figure 3:**
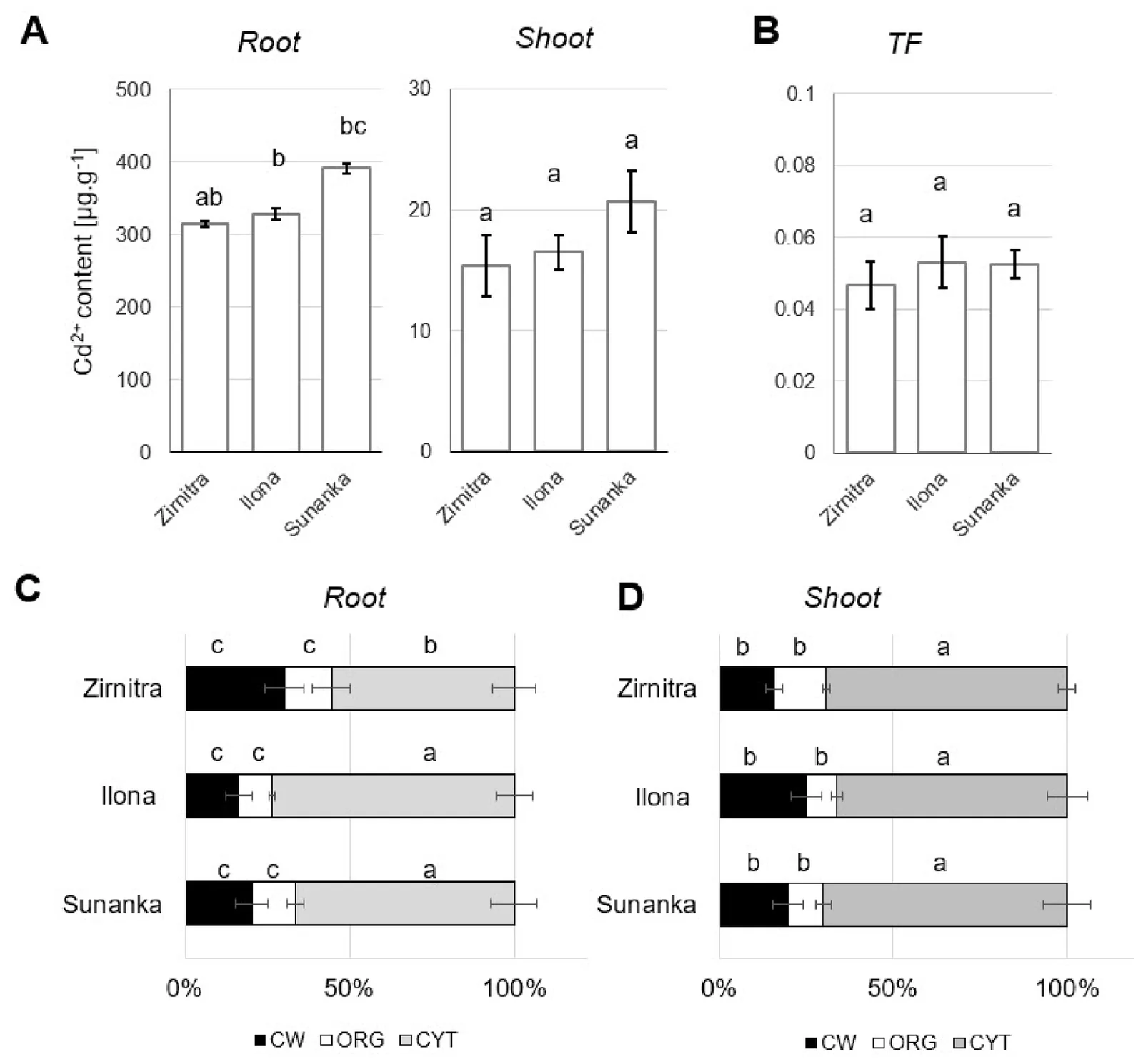
Cadmium accumulation, translocation, and subcellular distribution in three wheat cultivars. (**A**) Cd content in roots and shoots and (**B**) root-to-shoot translocation factor (TF). (**C**,**D**) Relative distribution of ^109^Cd among cell-wall (CW), organellar (ORG), and combined cytosolic/vacuolar (CYT) fractions in roots (**C**) and shoots (**D**). Data are means ± SD ((**A**,**B**); *n* = 3) or ± SE ((**C**,**D**); *n* = 3 biological replicates). Different letters indicate significant differences within the respective analyses (*p* < 0.05). For clarity, Cd concentrations in control plants are not shown in (**A**); however, they were included in the statistical analysis and all belonged to Duncan group “a”.

Cd accumulated predominantly in roots, with considerably lower concentrations detected in shoots (Figure 3A). Root Cd concentrations were highest in Sunanka, significantly exceeding both Zirnitra and Ilona; Ilona and Zirnitra also differed significantly from each other. Similarly, Sunanka had the highest shoot Cd concentration and significantly exceeded both Ilona and Zirnitra (Figure 3A). Translocation transfer factors (TFs) ranged from 0.045 to 0.056 without significant differences among cultivars (Figure 3B), demonstrating limited root-to-shoot Cd translocation in all three cultivars. Two-way ANOVA further revealed significant cultivar × Cd interactions for Cd accumulation in both roots and shoots (Supplementary Data S1).

Subcellular fractionation revealed that the cytosolic/vacuolar (CYT) fraction accounted for the majority of accumulated Cd in both roots (55.6–73.6%) and shoots (66.2–70.1%), with the remainder distributed between the cell wall (CW, 15.7–30.3%) and organellar (ORG, 8.8–15.0%) fractions (Figure 3C,D). In roots, the CYT proportion was significantly lower in Zirnitra than in Ilona and Sunanka, which did not differ significantly from each other. In shoots, the CYT proportion did not differ significantly among cultivars. Neither in roots nor in shoots did the CW and ORG fractions differ significantly, either between cultivars or from each other within a cultivar, indicating that cultivar-specific differences in subcellular Cd partitioning were driven primarily by the CYT fraction rather than by differences in cell wall binding or organellar sequestration.

### 3.3. Cd Alters Polyphenol Profiles in a Tissue- and Cultivar-Specific Manner

**Figure 4:**
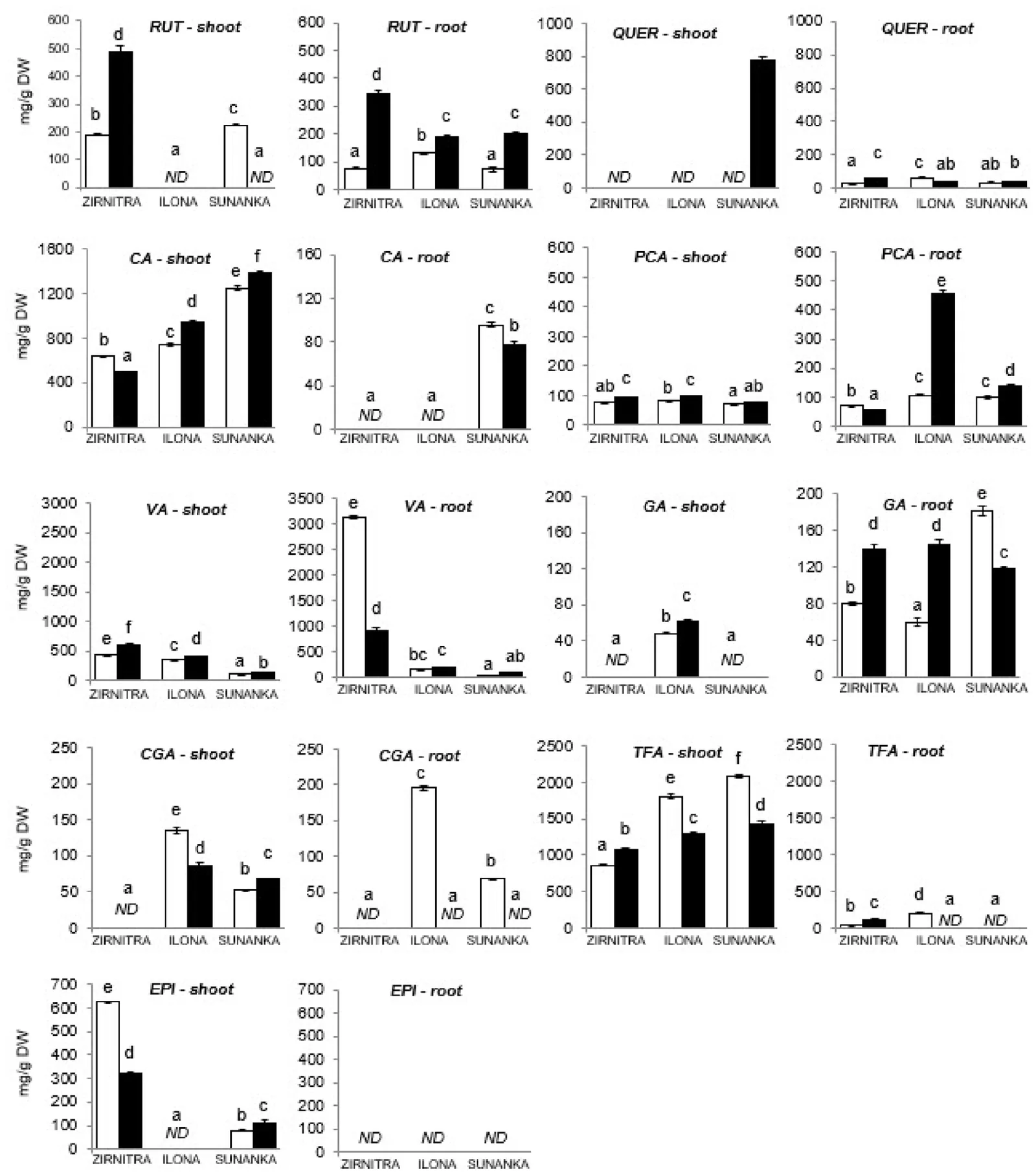
Effect of Cd treatment on phenolic metabolite profiles in shoots and roots of three wheat cultivars. Concentrations of individual phenolic metabolites in shoots (left panels) and roots (right panels) under control and Cd treatment. RUT, rutin; QUER, quercetin; EPI, epicatechin; CA, caffeic acid; CGA, chlorogenic acid; TFA, trans-ferulic acid; GA, gallic acid; PCA, protocatechuic acid; VA, vanillic acid. White bars represent control plants, and grey bars represent Cd-treated plants. Data are means ± SD (*n* = 3 biological replicates). Different letters indicate significant differences among cultivar × treatment combinations (one-way ANOVA followed by Duncan’s multiple range test, *p* < 0.05). ND, not detected.

Polyphenol profiling revealed marked tissue- and cultivar-specific differences in flavonoid accumulation and responses to Cd treatment (Figure 4).

#### 3.3.1. Flavonoid Accumulation: Epicatechin, Rutin, and Quercetin

Rutin (RUT) represents the glycosylated form (quercetin-3-rutinoside) of the quercetin aglycone (QUER). In Sunanka shoots, RUT was not detected following Cd treatment, whereas QUER increased from undetectable levels in controls to the highest concentration recorded among all analysed groups. In Sunanka roots, however, both compounds increased simultaneously (Figure 4). Cd stress markedly induced RUT in both shoots and roots of Zirnitra, while QUER remained undetectable in shoots under both conditions. In roots, however, QUER also increased significantly. Interestingly, Ilona was distinguished by the absence of both compounds in shoots (not detected in either control or Cd-treated plants) and divergent root responses.

Epicatechin (EPI) was detected only in shoots of Sunanka and Zirnitra and was not detected in roots of any cultivar. Zirnitra showed the highest baseline EPI level but a significant depletion under Cd stress. Sunanka responded to Cd with a significant, moderate elevation of EPI level. In Ilona, EPI was not detected in either control or Cd-treated plants (Figure 4).

#### 3.3.2. Hydroxycinnamic Acids

Hydroxycinnamic acids—caffeic acid (CA), chlorogenic acid (CGA) and trans-ferulic acid (TFA)—showed pronounced cultivar-specific responses under Cd stress (Figure 4). TFA was among the most abundant hydroxycinnamic acids in shoots, declining in Ilona and Sunanka shoots but increasing in Zirnitra in response to Cd. In roots, TFA level increased markedly in Zirnitra, but dropped below detection limit in Ilona, while it was not detected in Sunanka roots under either treatment. Caffeic acid was predominantly shoot-associated, with Zirnitra the only cultivar showing a decrease while Ilona and Sunanka increased. Root CA was not detected in Ilona or Zirnitra, while in Sunanka it was present and decreased significantly following Cd treatment. CGA was not detected in Zirnitra tissues. Cd stress caused divergent accumulation in shoots and complete depletion (not detected after treatment) in roots of both Ilona and Sunanka (Figure 4).

#### 3.3.3. Hydroxybenzoic Acids

Hydroxybenzoic acids—gallic acid (GA), protocatechuic acid (PCA), and vanillic acid (VA)—were predominantly root-associated (Figure 4). GA was detected in the roots of all three cultivars. Under Cd stress, root GA increased in Zirnitra and Ilona but declined in Sunanka, despite Sunanka having the highest baseline concentration. GA was additionally detected in Ilona shoots, where it increased significantly under Cd stress. PCA was the only compound present in all tissues and cultivars under both control and Cd conditions; in shoots, PCA increased significantly in all three cultivars, while in roots the response was highly divergent—Ilona showed an approximately four-fold increase, Sunanka increased moderately, and Zirnitra declined. VA was characterised by a markedly higher baseline in Zirnitra roots (compared to Ilona and Sunanka), which decreased significantly under Cd stress yet remained the highest root value across all cultivars; in contrast, root VA increased in Ilona and more strongly in Sunanka, while in shoots, VA increased following Cd treatment in all three cultivars (Figure 4).

Significant cultivar × Cd interactions were detected for most phenolic metabolites in both roots and shoots (Supplementary Data S1), supporting cultivar-dependent differences in their responses to Cd exposure.

### 3.4. Cd Treatment Differentially Alters Chitinase and β-1,3-Glucanase Activities

**Figure 5:**
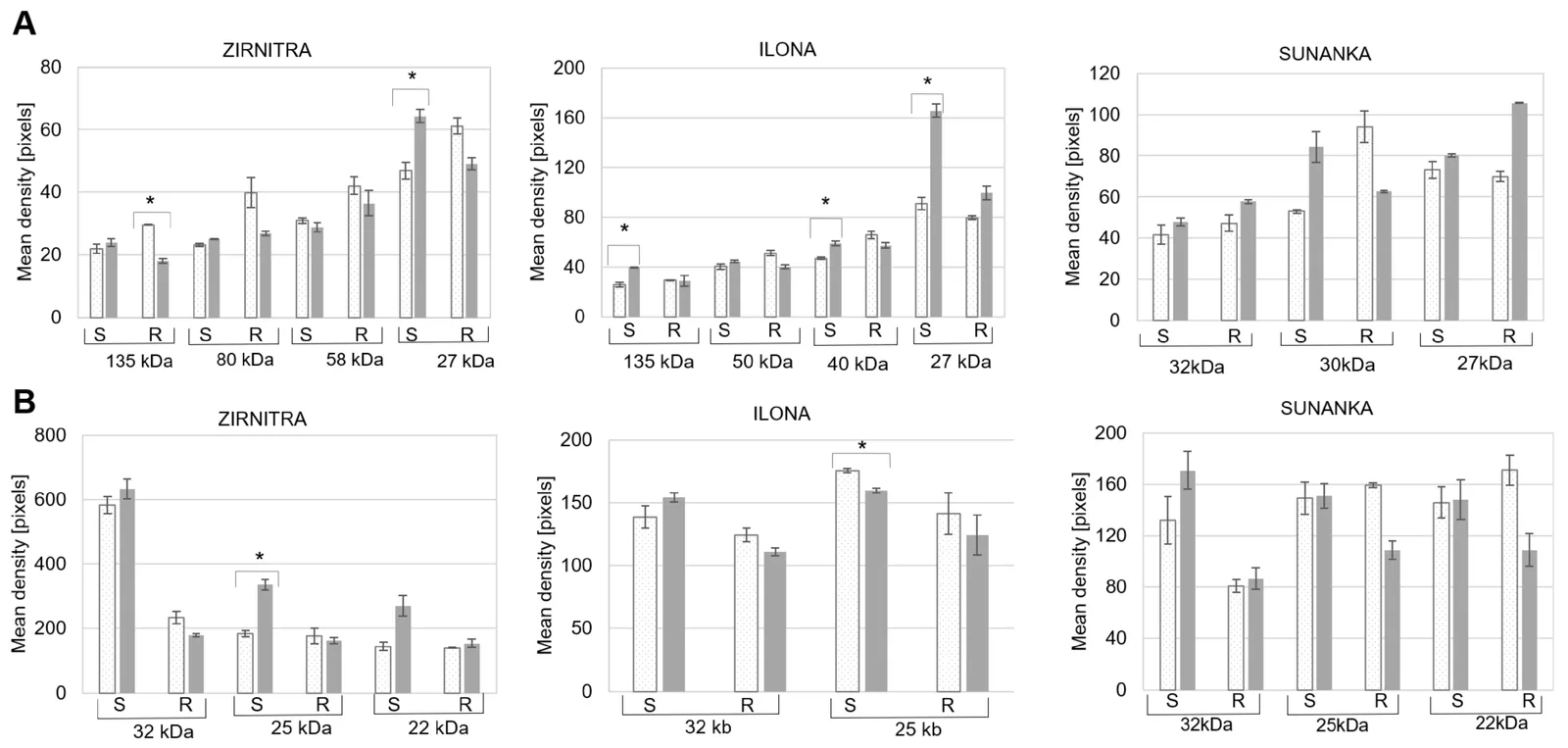
Effect of Cd treatment on PR-enzyme activities in shoots and roots of three wheat cultivars. Activities of chitinases (**A**) and β-1,3-glucanases (**B**) in shoots (S) and roots (R) under control (white bars) and Cd treatment (grey bars). Activity bands are designated by their apparent molecular masses (kDa). Data are means ± SD (*n* = 3 biological replicates). Asterisks indicate significant differences between control and Cd treatment within each cultivar, tissue, and activity band (Welch’s *t*-test with Benjamini–Hochberg FDR correction; q < 0.05).

Three chitinase activity bands with apparent molecular masses of approximately 32, 25, and 22 kDa were resolved by SDS-PAGE zymography, with the 22 kDa band not detected in either tissue of Ilona (Figure 5, Supplementary Figure S1). In shoots, Zirnitra was distinguished by significantly increased activity of the 25 kDa band, while Ilona showed a decrease in the 25 kDa band and the 32 kDa band remained unaffected across all cultivars. In roots, the observed changes were not significant.

Eight different β-1,3-glucanase activity bands were resolved by SDS-PAGE (Figure 5, Supplementary Figure S1), with a pronounced cultivar-dependent distribution: the 27 kDa band was detected in all three cultivars, the 135 kDa band was shared between Ilona and Zirnitra, while the remaining bands were each restricted to a single cultivar (80 and 58 kDa in Zirnitra; 50 and 40 kDa in Ilona; 32 and 30 kDa in Sunanka). A general tissue-dependent pattern was observed, with most responsive shoot bands increasing under Cd treatment, whereas several root bands decreased. In shoots, significant increases were observed for the 27 kDa band in Ilona and Zirnitra, the 135 kDa band in Ilona, but none in Sunanka. In roots, significant decrease was recorded only for the 135 kDa band in Zirnitra. The 27 kDa band activity increased significantly in Ilona, displaying a cultivar-specific activity response of this shared band (Figure 5). Significant cultivar × Cd interactions were also detected for β-1,3-glucanase activity bands at 135, 80, 50, 40, 30, and 27 kDa and chitinase activity bands at 32 and 22 kDa in roots (Supplementary Data S1).

### 3.5. Principal Component and Correlation Analyses Reveal Distinct Cultivar-Specific Biochemical Profiles

**Figure 6:**
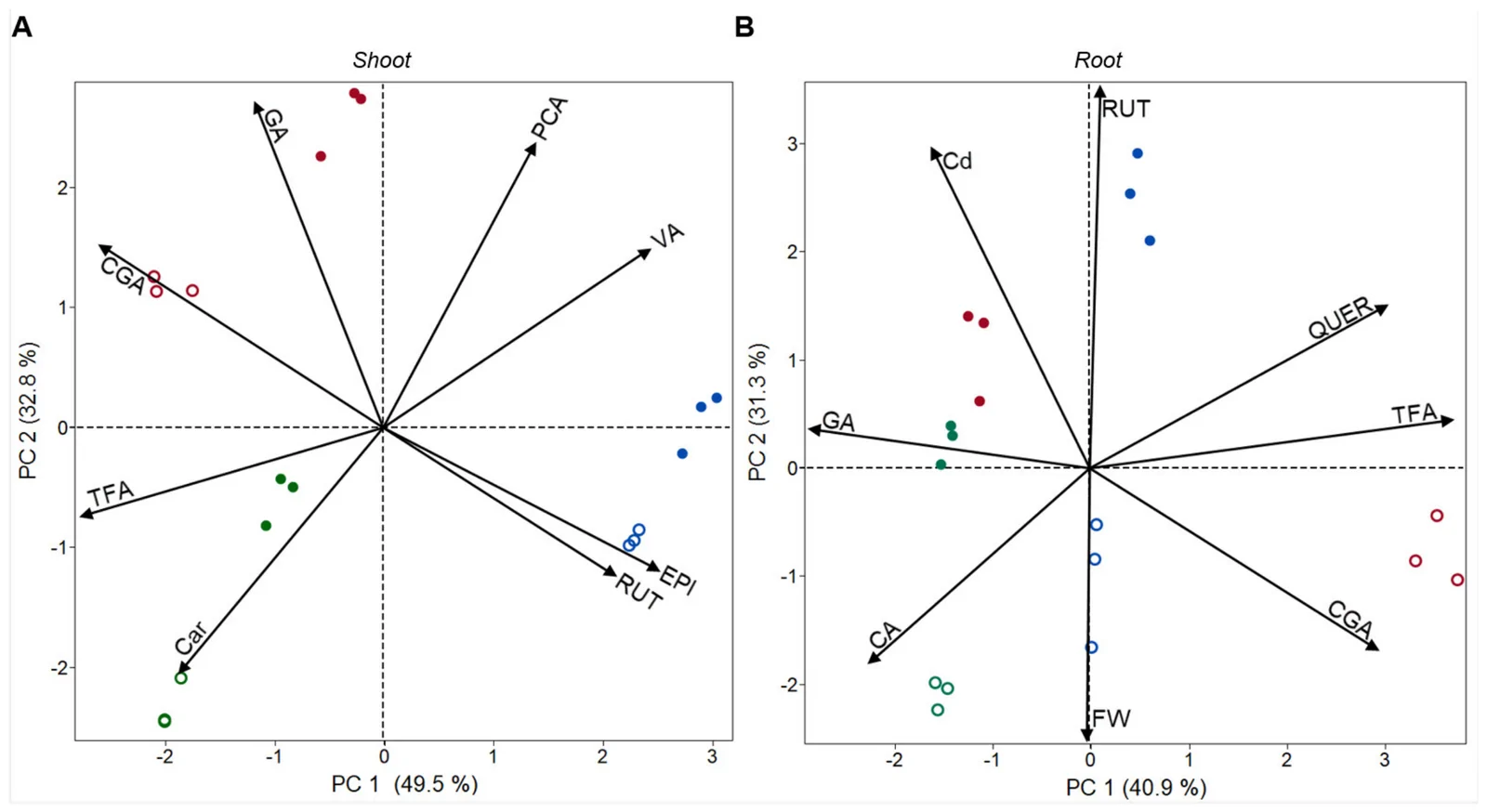
Principal component (PC) analysis of physiological and phenolic responses to Cd in three wheat cultivars. Biplots for shoots/shoots (**A**) and roots (**B**) under control and Cd treatment. Vectors represent physiological parameters and phenolic metabolites. Blue, green, and red symbols represent Zirnitra, Sunanka, and Ilona, respectively; open symbols indicate controls and filled symbols Cd-treated samples. Only informative variable loadings are displayed for clarity.

Principal component analysis of the shoot dataset (82.3% cumulative variance) showed that cultivar separation was largely associated with PC1, while treatment-related separation within cultivars was more strongly reflected along PC2 (Figure 6A). Each cultivar moved away from its characteristic control-associated metabolic profile—Ilona from chlorogenic acid, Zirnitra from epicatechin, Sunanka from carotenoids—consistent with the corresponding significant decreases in these compounds reported above (all *p* < 0.01).

Spearman correlation analysis (FDR-corrected; Table 1) identified contrasting associations of VA and TFA with pigment and PR-protein parameters. VA was negatively correlated with chlorophylls and carotenoids and positively correlated with chitinase activities, whereas TFA was positively correlated with chlorophylls and carotenoids and negatively correlated with the 32-kDa chitinase.

In roots (72.2% cumulative variance), cultivars were again separated largely along PC1 under control conditions (Figure 6B). Under Cd, Ilona showed a pronounced displacement, moving away from the TFA/CGA/QUER-associated region—consistent with TFA becoming undetectable and with the decreases in CGA and QUER—while Zirnitra and Sunanka shifted to a smaller extent, mainly toward RUT and GA, respectively. Cadmium content and root fresh weight vectors were oriented in near-opposite directions along PC2. However, Cd content and root FW were not significantly correlated in the FDR-corrected Spearman analysis (*ρ* = −0.108, *q* = 0.936). In roots, FDR-corrected correlations further revealed a positive association between Cd content and RUT, while RUT was also positively correlated with QUER (Table 1). However, the association between root Cd content and RUT was no longer statistically significant after adjustment for cultivar and treatment (*p* = 0.195; Supplementary Figure S2), suggesting that the pooled correlation was substantially influenced by differences among experimental groups. Among metabolite–PR-protein relationships, PCA was strongly positively correlated with the 27-kDa β-1,3-glucanase and negatively correlated with the 25-kDa chitinase (*ρ* = −0.633, *q* = 0.036). The 27-kDa β-1,3-glucanase was likewise negatively correlated with the 25-kDa chitinase, whereas VA was strongly positively correlated with the 32-kDa chitinase (Table 1).

Hierarchical cluster analysis broadly supported the principal component analysis patterns (Supplementary Figure S3). In shoots, control and Cd-treated samples retained strong cultivar-specific clustering, whereas root profiles showed a marked treatment-related reorganization. Notably, Ilona control and Cd-treated samples were assigned to widely separated branches, consistent with the pronounced Cd-induced displacement observed in the root PC analysis.

## 4. Discussion

The present study revealed that the three wheat cultivars shared a strong capacity to restrict Cd translocation from roots to shoots and showed broadly similar growth responses, yet differed markedly in their biochemical responses to Cd. This divergence was already evident in the photosynthetic pigment response. Cd-induced chlorophyll loss is well documented in wheat [1,2]; however, despite comparable reductions in shoot fresh weight (20–25%), chlorophyll content declined significantly in Sunanka and Ilona but remained stable in Zirnitra (Figure 2). Thus, comparable growth inhibition was associated with contrasting effects on the photosynthetic pigment pool.

The contrasting pigment responses occurred despite a broadly similar whole-plant pattern of Cd allocation. All three cultivars strongly restricted Cd translocation to the shoots, with low and comparable TF values, although Sunanka accumulated significantly more Cd in both roots and shoots. Preferential Cd retention in wheat roots has been associated with structural and cellular barriers that restrict root-to-shoot transport and thereby reduce Cd exposure of aboveground tissues [7]. Possible mechanisms contributing to this retention include Cd binding to root cell wall components and intracellular sequestration, potentially involving vacuolar compartmentalization [4,7,34]. However, these mechanisms were not directly investigated in the present study.

At the subcellular level, Cd accumulated predominantly in the combined cytosolic/vacuolar fraction, consistent with previous observations across wheat cultivars, including the cultivars studied here [9]. This distribution is compatible with intracellular Cd sequestration as an important detoxification mechanism [4,34], although our fractionation did not distinguish cytosolic from vacuolar pools. Intracellular Cd sequestration may involve binding to thiol-containing ligands, particularly phytochelatins and glutathione, followed by transport of Cd–ligand complexes into vacuoles. However, the predominance of Cd in the combined cytosolic/vacuolar fraction does not establish its precise subcellular localization or the contribution of individual sequestration pathways [4,11,34]. Cultivar differences were limited to a lower CYT proportion in Zirnitra than in Ilona roots, with no significant differences in CW or ORG fractions. Given the single experimental run, this cultivar-specific difference should be interpreted cautiously.

Cd and other heavy metals have been shown to induce chitinases and β-1,3-glucanases as part of a broader plant defence response [15]. In our study, Cd affected their activities in a cultivar-, tissue-, and activity-band-dependent manner. These contrasting responses may reflect differential regulation of stress-related defence pathways, as chitinases and β-1,3-glucanases, although primarily associated with pathogen defence, can also respond to abiotic stress. Their biological significance may extend to processes involving cell wall modification and stress signalling [21,23,25]. In addition, β-1,3-glucanases may contribute to the regulation of symplastic transport through plasmodesmata by mediating callose degradation [24]. The distinct activity-band patterns suggest that chitinase and β-1,3-glucanase activities are not uniformly regulated across cultivars and tissues. However, the functional significance of individual activity bands remains unclear, and their responses cannot be directly linked to Cd detoxification or tolerance based on the present data. Notably, Zirnitra showed clear changes in several PR-enzyme activities despite maintaining stable chlorophyll levels, indicating that pigment stability did not reflect an absence of biochemical stress. The absence of a consistent relationship between PR-enzyme activities and Cd accumulation (Table 1), also reported in other cereals [22,25], further supports their interpretation as markers of stress/defence activation rather than direct indicators of Cd detoxification.

The cultivar-specific biochemical response to Cd was particularly evident in phenolic metabolism. Rather than uniformly increasing phenolic accumulation, Cd differentially affected individual branches of the phenolic network, resulting in distinct tissue- and cultivar-dependent profiles [4,35,36]. One of the clearest examples of this reorganization occurred within the flavonol branch. In Sunanka shoots, RUT became undetectable under Cd treatment, coinciding with marked accumulation of its aglycone QUER, which reached the highest concentration among the analysed shoot samples. Because glycosylation can strongly affect flavonol stability, subcellular localization, and antioxidant properties [37], this reciprocal response is consistent with altered flavonol glycosylation status under Cd stress; however, endpoint metabolite concentrations cannot distinguish among changes in glycosylation, deglycosylation, synthesis, and turnover. A contrasting flavonol response occurred in Zirnitra roots, where RUT increased to approximately 4.5 times the control level. Although root Cd accumulation was positively associated with RUT content in the pooled dataset, this relationship was no longer statistically significant after adjustment for cultivar and treatment (*p* = 0.195; Supplementary Figure S2). The observed association should therefore be interpreted cautiously, as it may be substantially influenced by differences among experimental groups. RUT has previously been implicated in plant responses to Cd, as exogenous RUT alleviated Cd-induced oxidative damage and modified Cd accumulation and antioxidant responses in *Zygophyllum fabago* [38]. Root RUT accumulation may reflect antioxidant responses to Cd exposure, as suggested by previous findings on exogenous RUT application [38], although potential interactions with metal ions cannot be excluded. However, the observed Cd–RUT association does not demonstrate direct Cd binding, sequestration, or enhanced tolerance. Targeted manipulation of RUT metabolism, combined with Cd localization and binding analyses, would be required to establish its functional significance. However, despite the broader involvement of phenylpropanoid metabolism in root responses to Cd [39], Cd-related changes in endogenous RUT and their relationship to tissue Cd accumulation appear to remain poorly characterized, particularly in wheat. Root RUT accumulation may reflect antioxidant responses to Cd exposure, although potential interactions with metal ions cannot be excluded. Nevertheless, the observed association does not demonstrate direct Cd binding, sequestration, or enhanced tolerance. Targeted manipulation of RUT metabolism, combined with Cd localization and binding analyses, would be required to establish its functional significance. A second, distinct reorganization was evident in the hydroxycinnamate/hydroxybenzoate pools, most prominently in Ilona roots, where *trans*-ferulic acid (TFA) became undetectable and chlorogenic acid (CGA) declined sharply, while protocatechuic acid (PCA) accumulated to approximately four times its control level. Previous studies have likewise demonstrated Cd-induced changes in phenolic metabolism in wheat. Cd exposure was associated with activation of phenylpropanoid metabolism and increased caffeic acid (CA) and ferulic acid levels [19], whereas targeted profiling revealed compound-specific changes in several phenolic acids, including CA and CGA, depending on Cd concentration and experimental conditions [18]. The contrasting responses observed here further indicate that Cd-induced modulation of phenylpropanoid metabolism does not necessarily result in parallel changes in individual phenolic metabolites. The phenolic network in wheat comprises multiple interconnected biosynthetic branches whose differential regulation can generate distinct metabolite profiles [16,40]. The reciprocal TFA/CGA–PCA pattern observed in Ilona roots is therefore consistent with differential regulation of these interconnected phenolic branches, including potential benzoate-forming routes derived from phenylpropanoid intermediates [41], but does not demonstrate direct metabolic conversion, since pathway fluxes were not measured. GA showed a different pattern, increasing in Zirnitra and Ilona roots but declining in Sunanka. Its distinct response may partly reflect its biosynthetic origin within the shikimate pathway, outside the classical phenylalanine-derived phenylpropanoid route [17]. Together, these contrasting responses indicate differential engagement of interconnected phenolic branches rather than a coordinated shift in the entire phenolic pool.

**Figure 7:**
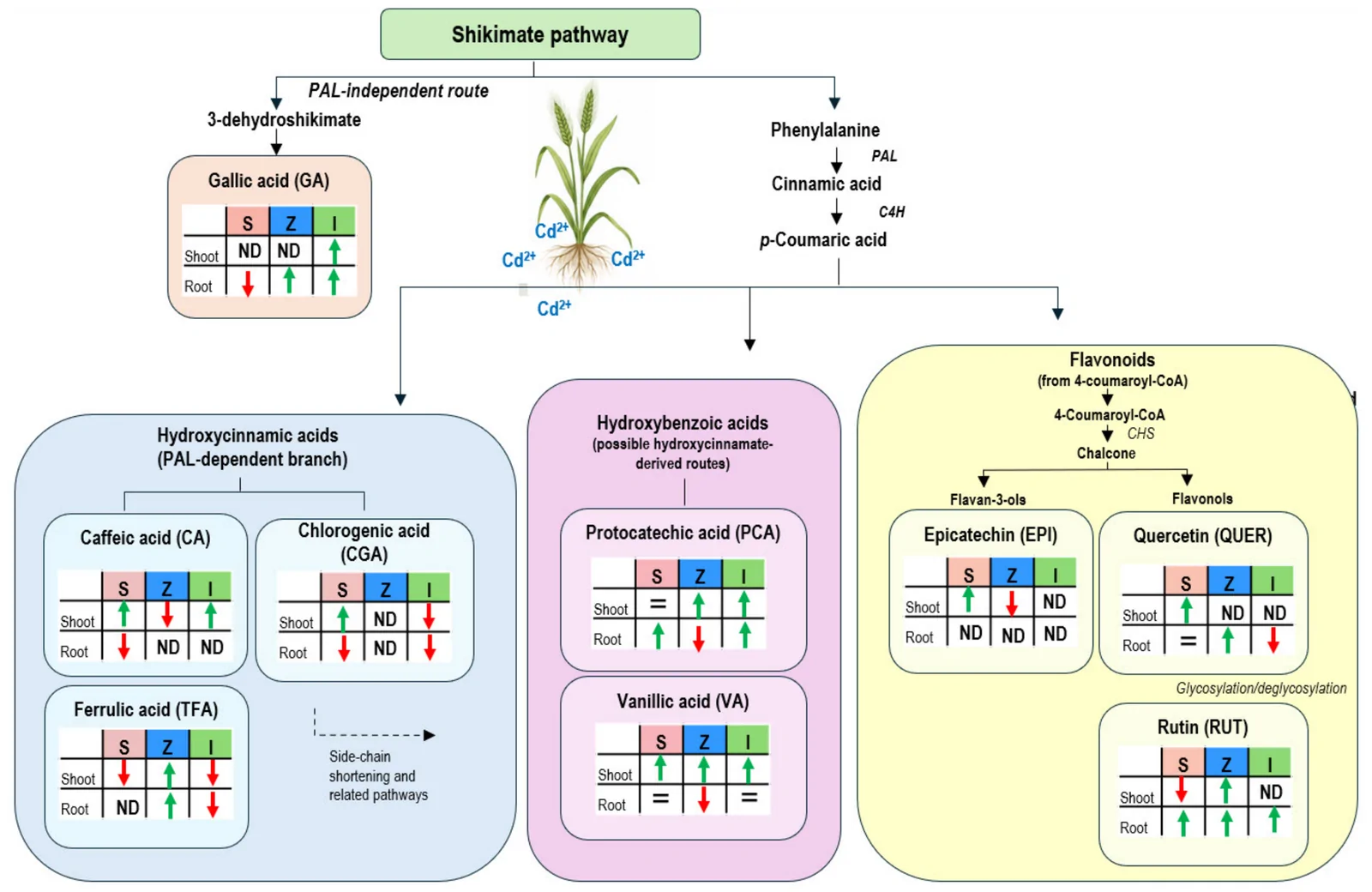
Schematic overview of cultivar-specific changes in phenolic metabolites in three wheat cultivars in response to Cd treatment. The scheme shows the major metabolic relationships among the analysed phenolic compounds. Symbols indicate significant increases (↑), decreases (↓), or no significant changes (=) relative to the corresponding control; ND indicates compounds not detected under either condition. Changes from or to non-detectable levels are indicated by ↑ or ↓, respectively. S, Sunanka; Z, Zirnitra; I, Ilona. Dashed arrows indicate simplified or possible metabolic routes rather than direct conversions.

These branch-specific responses were reflected in the multivariate structure of the metabolite data (Figure 7). In shoots, the strong cultivar separation suggests that Cd-associated changes occurred against distinct baseline metabolic profiles, whereas root profiles revealed pronounced cultivar-dependent differences in metabolic responses to Cd. This was particularly evident in Ilona, characterized by depletion of the TFA/CGA/QUER-associated profile, while Zirnitra was distinguished by strong root RUT accumulation and Sunanka by a shift away from its CA-associated control profile, accompanied by pronounced increases in VA and RUT. Collectively, these patterns indicate that Cd modified pre-existing cultivar-specific phenolic configurations rather than driving the three cultivars toward a common metabolic state, with HCA providing complementary visualization of these sample-grouping patterns (Supplementary Figure S3).

Importantly, this metabolic divergence occurred despite broadly comparable shoot growth inhibition and similarly restricted root-to-shoot Cd translocation, indicating that similar whole-plant outcomes were associated with distinct biochemical configurations. This is consistent with previous physiological and omics studies demonstrating strong genotype dependence of Cd responses in wheat [4,5,6]. Together, these findings suggest that comparable whole-plant responses to Cd may arise from different combinations of biochemical adjustments among genotypes.

Within this framework, the three cultivars displayed distinct biochemical configurations. Zirnitra combined pigment stability with pronounced root RUT accumulation and changes in PR-enzyme activities, whereas Sunanka showed the most pronounced relative pigment decline together with marked flavonol reorganization, and Ilona was characterized by pronounced restructuring of root hydroxycinnamate/hydroxybenzoate profiles. These findings extend previous evidence for Cd-responsive phenolic metabolism in wheat by showing that Cd-induced changes are strongly cultivar- and tissue-dependent and involve different components of the phenolic network rather than a uniform shift in phenolic metabolism. Whether these differences reflect broader contrasts between emmer and bread wheat germplasm or cultivar-specific variation cannot be resolved from the present three-cultivar comparison.

The present study provides an integrated perspective on physiological and phytochemical responses to Cd exposure in three wheat cultivars, highlighting both shared and cultivar-specific patterns. As the experiment involved a single Cd concentration (50 mg L^−1^) and one harvest time (10 days), the observed cultivar-specific responses cannot necessarily be extrapolated to other Cd exposure levels or durations. Although the experimental design does not resolve metabolic fluxes or establish causal relationships between biochemical changes and Cd tolerance, the observed patterns provide a basis for further mechanistic investigations. The limited replication of the subcellular fractionation experiment should also be considered when interpretating cultivar-specific Cd partitioning. Broader genotype screening combined with targeted analysis of phenylpropanoid pathway regulation could help establish whether the biochemical patterns identified here are conserved across wheat germplasm. Preliminary results from this study were previously presented at the AgroEkoBios 2026 conference and published as a conference abstract [42].

## 5. Conclusions

Comparable growth inhibition and similarly restricted root-to-shoot Cd translocation were accompanied by distinct biochemical responses among wheat cultivars, particularly in phenolic metabolism and PR-enzyme activities. These differences were observed both between the tetraploid emmer cultivar Zirnitra and the hexaploid bread wheat cultivars Sunanka and Ilona, and among the two hexaploid cultivars themselves, highlighting cultivar-dependent variation rather than a consistent ploidy-associated response. The association between root RUT accumulation and Cd burden across cultivars identifies RUT as a candidate for further investigation, but whether this relationship is functionally involved in the Cd response or merely reflects parallel stress-induced changes remains to be determined. Molecular identification of the responsive PR-enzyme activity bands could likewise clarify their contribution to cultivar-specific responses. Future studies combining these approaches with targeted manipulation of phenolic metabolism may help distinguish biochemical associations from mechanisms directly contributing to Cd responses in wheat.

## Figures and Tables

**Table 1:** Significant Spearman correlations between metabolite, mineral, and PR-protein activity parameters.

Variable 1	Variable 2	Spearman ρ	q (FDR)
Root			
Protocatechuic acid (PCA)	27-kDa β-1,3-glucanase	0.928	0.0022
Vanillic acid (VA)	32-kDa chitinase	0.893	0.0022
Rutin (RUT)	Cd content	0.734	0.0075
Quercetin (QUER)	Rutin (RUT)	0.664	0.0304
25-kDa chitinase	27-kDa β-1,3-glucanase	−0.633	0.0360
Protocatechuic acid (PCA)	25-kDa chitinase	−0.633	0.0360
Shoot			
Cd content	Fresh weight (FW)	−0.867	0.0010
Caffeic acid (CA)	25-kDa chitinase	−0.851	0.0010
Vanillic acid (VA)	Caffeic acid (CA)	−0.854	0.0010
Trans-Ferulic acid (TFA)	32-kDa chitinase	−0.802	0.0010
Trans-Ferulic acid (TFA)	Vanillic acid (VA)	−0.854	0.0010
Protocatechuic acid (PCA)	Total chlorophyll (Chl*a*+Chl*b*) ^a^	−0.719	0.0059
Vanillic acid (VA)	25-kDa chitinase	0.723	0.0057
Vanillic acid (VA)	Protocatechuic acid (PCA)	0.713	0.0059
Vanillic acid (VA)	32-kDa chitinase	0.674	0.0136
32-kDa chitinase	27-kDa β-1,3-glucanase	−0.659	0.0152
Trans-Ferulic acid (TFA)	Caffeic acid (CA)	0.649	0.0172
Trans-Ferulic acid (TFA)	Total chlorophyll (Chl*a*+Chl*b*) ^a^	0.633	0.0202
Caffeic acid (CA)	32-kDa chitinase	−0.626	0.0210
Vanillic acid (VA)	Total chlorophyll (Chl*a*+Chl*b*) ^a^	−0.614	0.0251

Only correlations that remained significant after Benjamini–Hochberg false discovery rate (FDR) correction (q < 0.05) and that involved variables detected in all three cultivars within the given tissue are shown. Correlations involving cultivar-restricted variables (including most zymography bands and several phenolic compounds) were excluded prior to correction, as their apparent covariation reflects the categorical structure of genotype grouping rather than biological association; the complete correlation analysis is provided in Supplementary Data S2. ^a^ Representative value shown; the corresponding metabolite was also significantly correlated with the individual chlorophyll and carotenoid components (Chl*a*, Chl*b*, Car), which are highly intercorrelated with each other (ρ = 0.92–0.99) and are therefore not listed separately (see Supplementary Data S2 for full values).

## Data Availability

The data supporting the findings of this study are openly available in the Zenodo repository at https://doi.org/10.5281/zenodo.23355428.

## Supplementary Materials

The following supporting information can be downloaded at: https://www.mdpi.com/article/10.3390/biology15211932/s1, Supplementary_Data_S1: Biological-replicate data matrices and ANOVA outputs, including data on ^109^Cd subcellular fractionation; Supplementary_Data_S2: Spearman correlation matrices and FDR correction results for roots and shoots; Supplementary_Figure_S1: Representative zymograms of PR-enzyme activities in shoots and roots of three wheat cultivars; Supplementary_Figure_S2: Rutin content in roots and shoots of three wheat cultivars under control and cadmium-treated conditions; Supplementary_Figure_S3: Hierarchical cluster analysis of physiological and phenolic responses to cadmium, complementing the principal component analysis presented in the manuscript. The Supplementary Materials file is available in the Zenodo repository at DOI: 10.5281/zenodo.23355428 and contains the following files.
