# Supplementary material for: Physiological and Phytochemical Responses to Cadmium Stress in Three Wheat Cultivars
Source: Biology (Basel). 2026 Oct 24;15(21):1932. doi: 10.3390/biology15211932 (PMC13761590; doi:10.3390/biology15211932)
Supplement: Supplementary file 1 [file biology-15-01932-s001.zip › Supplementary_Figure_S1_Zymograms.pdf]

Physiological and Phytochemical Responses to Cadmium Stress in Three Wheat

Laura Žideková, Anirban J. Debnath, Martina Gregorová, Peter Nemeček, Petra Ranušová, Monika Šutáková, Miroslava Sinčák, Pavol Hauptvogel, Ildikó Matušíková

This file contains two supplementary figures (Figures S1 and S2). The underlying research data associated with these supplementary materials and the main manuscript are available within the same Zenodo repository at ***DOI: 10.5281/zenodo.22742549.***

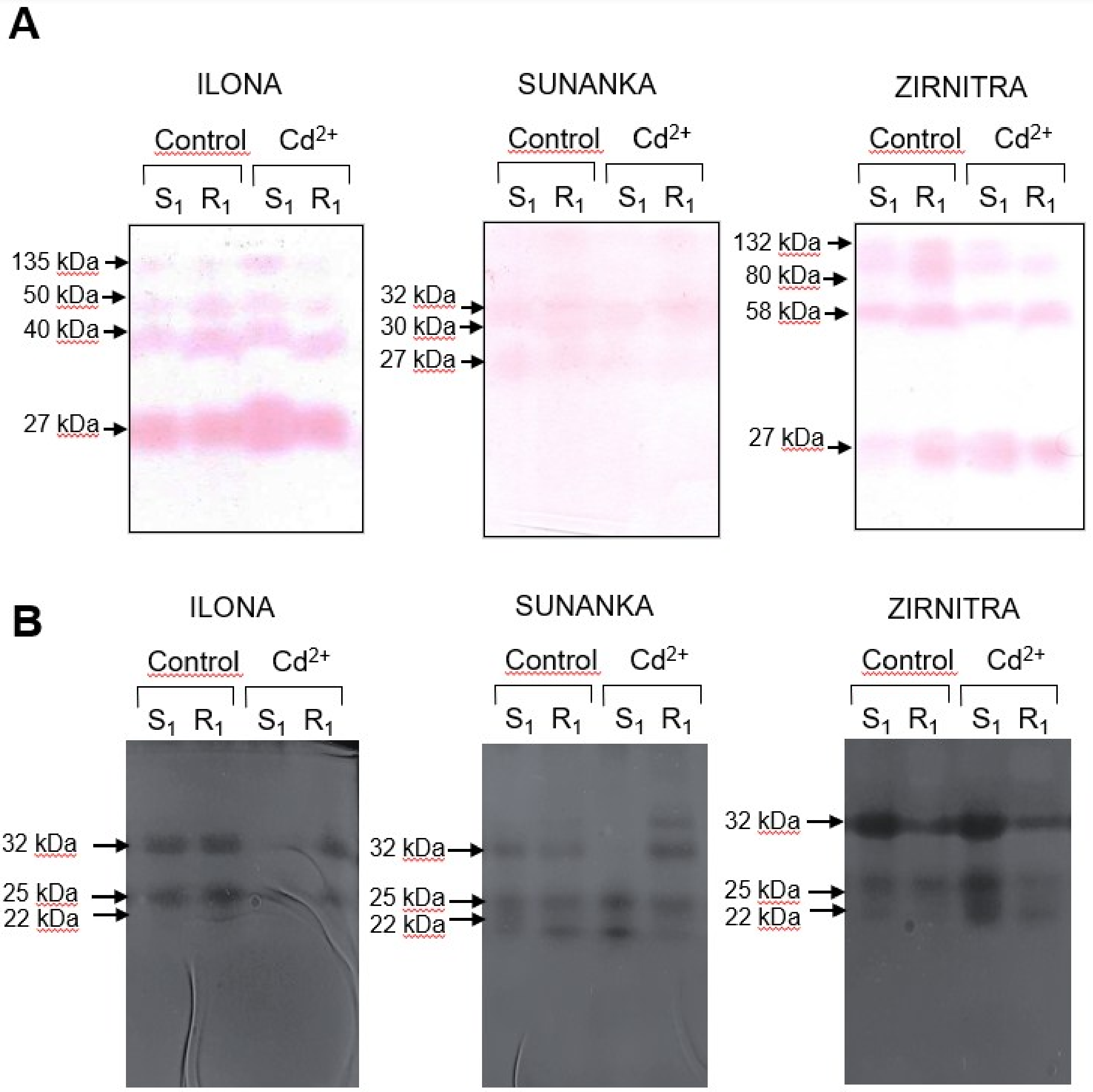

Supplementary Figure S1. Representative zymograms of PR-enzyme activities in shoots and roots of three wheat cultivars. (A) Chitinase and (B) β-1,3-glucanase activities in shoots (S) and roots (R) of Ilona, Sunanka, and Zirnitra under control and Cd treatment. Arrows indicate activity bands and their estimated apparent molecular masses (kDa). Apparent molecular masses were estimated using unstained (10–200 kDa) and prestained (11–245 kDa) protein molecular-weight standards.
