# Supplementary material for: Physiological and Phytochemical Responses to Cadmium Stress in Three Wheat Cultivars
Source: Biology (Basel). 2026 Oct 24;15(21):1932. doi: 10.3390/biology15211932 (PMC13761590; doi:10.3390/biology15211932)
Supplement: Supplementary file 1 [file biology-15-01932-s001.zip › Supplementary_Figure_S2_Rutin.pdf]

Physiological and Phytochemical Responses to Cadmium Stress in Three Wheat

Laura Žideková, Anirban J. Debnath, Martina Gregorová, Peter Nemeček, Petra Ranušová, Monika Šutáková, Miroslava Sinčák, Pavol Hauptvogel, Ildikó Matušíková

This file contains two supplementary figures (Figures S1 and S2). The underlying research data associated with these supplementary materials and the main manuscript are available within the same Zenodo repository at ***DOI: 10.5281/zenodo.22742549.***

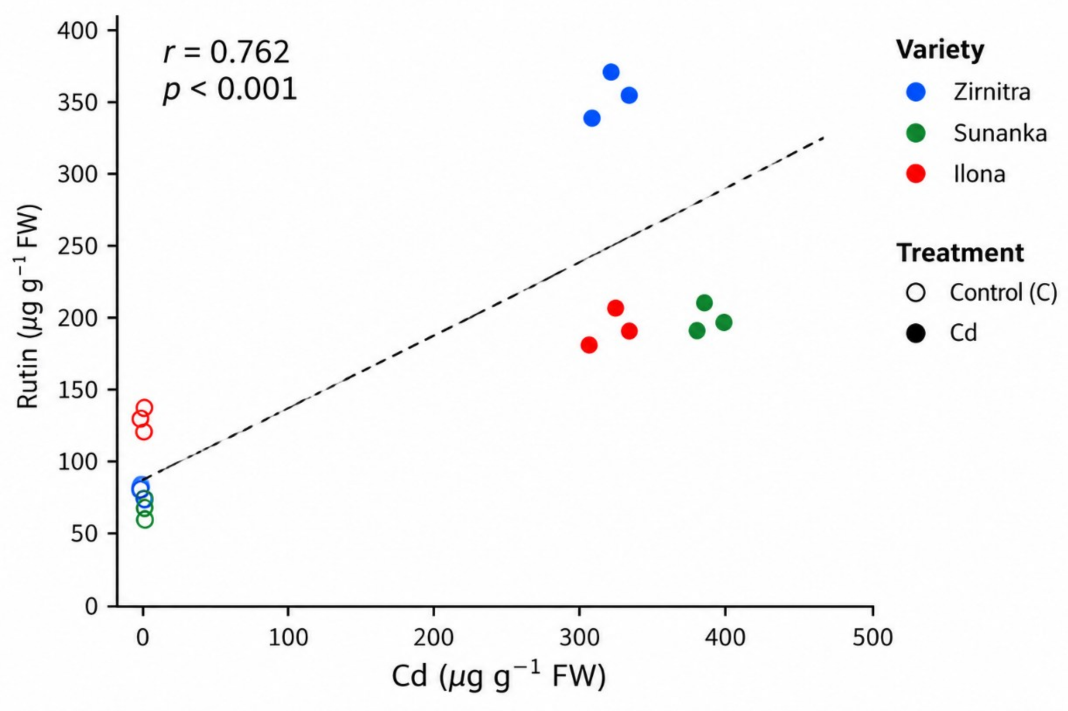

Supplementary Figure S2. Rutin content in roots and shoots of three wheat cultivars under control and cadmium-treated conditions. Relationship between root cadmium (Cd) accumulation and rutin content in three varieties (Zirnitra, Sunanka, and Ilona) under control and Cd-stress conditions. Each point represents an individual biological replicate (n = 3 per variety and treatment). Colors distinguish varieties, while symbols distinguish treatments. The dashed line represents the pooled linear regression across all observations (Pearson's r = 0.762, p < 0.001).
