# Supplementary material for: Physiological and Phytochemical Responses to Cadmium Stress in Three Wheat Cultivars
Source: Biology (Basel). 2026 Oct 24;15(21):1932. doi: 10.3390/biology15211932 (PMC13761590; doi:10.3390/biology15211932)
Supplement: Supplementary file 1 [file biology-15-01932-s001.zip › Supplementary_Figure_S3_HCA.pdf]

Physiological and Phytochemical Responses to Cadmium Stress in Three Wheat

Laura Žideková, Anirban J. Debnath, Martina Gregorová, Peter Nemeček, Petra Ranušová, Monika Šutáková, Miroslava Sinčák, Pavol Hauptvogel, Ildikó Matušíková

This file contains two supplementary figures (Figures S1 and S2). The underlying research data associated with these supplementary materials and the main manuscript are available within the same Zenodo repository at ***DOI: 10.5281/zenodo.22742549.***

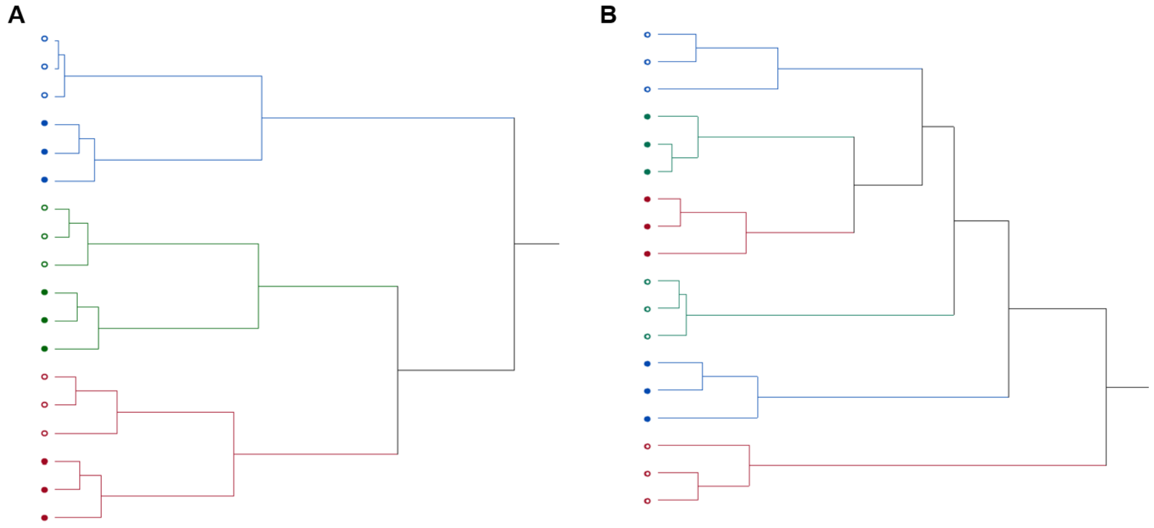

Supplementary Figure S3. Hierarchical cluster analysis (HCA) of physiological and phenolic responses to Cd in three wheat cultivars. HCA of leaf/shoot (A) and root (B) profiles under control and Cd treatment, using Ward's clustering method and squared Euclidean distance. Blue, green, and red indicate Zirnitra, Sunanka, and Ilona, respectively; open symbols represent controls and filled symbols Cd-treated samples. HCA was performed using the same physiological and phenolic variables as the corresponding PCA analyses.
